# A key gene for the climatic adaptation of *Apis cerana* populations in China according to selective sweep analysis

**DOI:** 10.1186/s12864-023-09167-x

**Published:** 2023-03-06

**Authors:** Yi Zhang, Hao Xu, Zhi Wang, Haoliang Jie, Fuchao Gao, Minqi Cai, Kang Wang, Dafu Chen, Rui Guo, Zheguang Lin, Qingsheng Niu, Ting Ji

**Affiliations:** 1 College of Animal Science and Technology, Yangzhou University, Yangzhou, 225009 China; 2 Sericultural Research Institute, Anhui Academy of Agricultural Science, Hefei, 230061 China; 3 Apiculture Science Institute of Jilin Province, Jilin, 132108 China; 4 Jinzhong Agriculture and Rural Affairs Bureau, Jinzhong, 030601 China; 5 Mudanjiang Branch of Heilongjiang Academy of Agricultural Sciences, Mudanjiang, 157043 China; 6 College of Animal Science (College of Bee Science), Fujian Agriculture and Forestry University, Fuzhou, 350002 China

**Keywords:** *Apis cerana*, Adaptive radiation distribution, Climate change, Population genomics, Selective sweep analysis

## Abstract

**Background:**

*Apis cerana* is widely distributed in China and, prior to the introduction of western honeybees, was the only bee species kept in China. During the long-term natural evolutionary process, many unique phenotypic variations have occurred among *A. cerana* populations in different geographical regions under varied climates. Understanding the molecular genetic basis and the effects of climate change on the adaptive evolution of *A. cerana* can promote *A. cerana* conservation in face of climate change and allow for the effective utilization of its genetic resources.

**Result:**

To investigate the genetic basis of phenotypic variations and the impact of climate change on adaptive evolution, *A. cerana* workers from 100 colonies located at similar geographical latitudes or longitudes were analyzed. Our results revealed an important relationship between climate types and the genetic variation of *A. cerana* in China, and a greater influence of latitude compared with longitude was observed. Upon selection and morphometry analyses combination for populations under different climate types, we identified a key gene RAPTOR, which was deeply involved in developmental processes and influenced the body size.

**Conclusion:**

The selection of RAPTOR at the genomic level during adaptive evolution could allow *A. cerana* to actively regulate its metabolism, thereby fine-tuning body sizes in response to harsh conditions caused by climate change, such as food shortages and extreme temperatures, which may partially elucidate the size differences of *A. cerana* populations. This study provides crucial support for the molecular genetic basis of the expansion and evolution of naturally distributed honeybee populations.

**Supplementary Information:**

The online version contains supplementary material available at 10.1186/s12864-023-09167-x.

## Background

Over the past few decades, the numbers of many insects have shown a rapid decline [1], including those of bumble bees [2], caterpillars [3], beetles [4], and honeybees [5]. The sources of insect survival pressure leading to these declines were abundant and included invasive species, habitat loss, pesticide abuse, environmental pollution, and climate change [6]. Of these factors, climate change, or its combination with all other factors, is more likely to be the main cause of large-scale global insect population and diversity declines [7–12]. However, evolutionary adaptations can potentially help species cope with survival pressure brought about by climate change [13]. *Apis cerana* is an important crop-pollinating insect in China. Its long collection cycle and ability to utilize sporadic nectar sources have enabled *A. cerana* to play a key role in the development of Chinese agriculture. During the long-term natural evolutionary process, many unique phenotypic variations have occurred among *A. cerana* populations in different regions under different climatic conditions [14, 15]. In general, moving from south to north in geographical distribution, the phenotype of *A. cerana* gradually shows a trend of weakening migration, slightly larger size, darker body color, and stronger cold resistance [16]. The characteristics of this adaptive radiation distribution under different climatic conditions show exceptional stress resistance, making *A. cerana* an excellent research subject for the study of climatic adaptation on adaptive evolution [17]. Thus, the study of the climatic adaptation of *A. cerana* can help effectively conserve *A. cerana* against climate change challenges. Simultaneously, understanding the genetic basis of these unique phenotypic variations is beneficial for their conservation and provides insight to better harness their genetic resources.

So far, many studies have used different methods to elucidate the basis of the unique phenotypic differences in *A. cerana*. Based on direct measurements of the phenotypic and morphological characteristics of *A. cerana* in different regions [18], nine varieties were classified. With the development of molecular biology technology, population genetics studies revealing genetic diversity based on sequencing the individual genomes of different bee species have been widely used [19–21]. Once the genome of *A. cerana* was published in 2015 (GCA_001442555.1) [22], genome research entered a rapid stage (GCA_002290385.1 and GCA_011100585.1) [23, 24]. Studies on the origin and evolution [14, 15, 17, 18, 25] and sequencing of *A. cerana* with special traits—*A. cerana abansis* (Aba strain) [26] and the endemic *A. cerana* with cold resistance [27]—progressed rapidly. These studies have also demonstrated distinct variations in DNA and microsatellites among *A. cerana* from different regions. A 2020 study identified the role of the leucokinin receptor (Lkr) in influencing the foraging division of labor [18]. However, existing research is far from sufficient to fully elucidate the climatic adaptation during the evolutionary process of *A. cerana*.

In this study, we collected 100 samples from 10 regions in China (covering the most important climate types) and utilized population genetics methods to identify the key traces in the process of adaptation to different environments at the genome level; we aimed to reveal the genetic basis of the strong environmental adaptability of *A. cerana*. Simultaneously, we chose collection sites at similar latitudes or longitudes to explore the influence of geographical coordinate factors on the adaptive radiation of *A. cerana*. Upon genetic structure, genetic differentiation, and population diversity analyses of these 10 populations, as well as through selective sweep analysis and morphometry analysis combination of the populations with high genetic differentiation, we were able to identify the key signaling pathways and genes involved in the process of adaptation to different climatic conditions in China. This study provides a useful reference for the improvement of *A. cerana* conservation and breeding.

## Results

### Sampling and sequencing

**Fig. 1:**
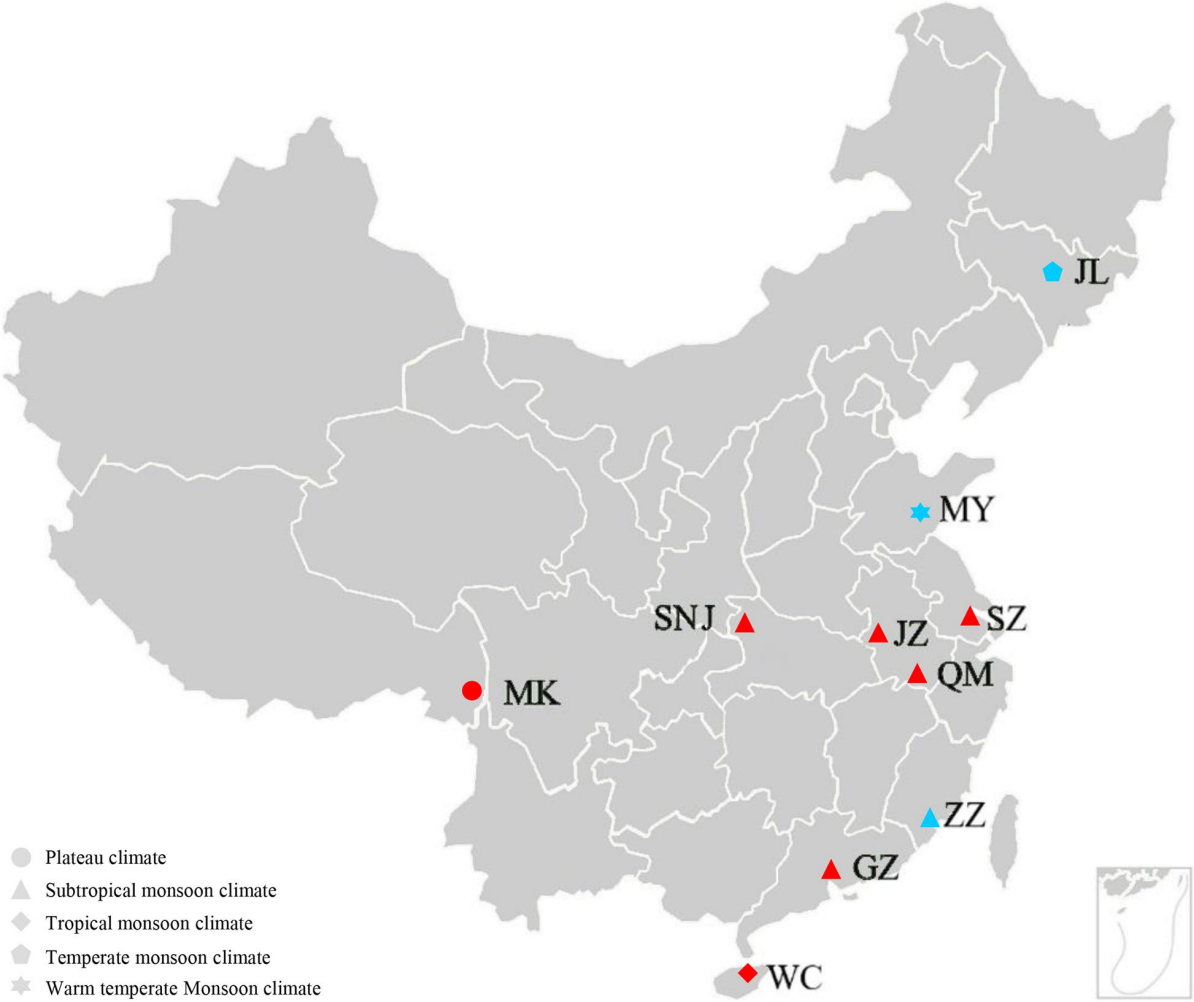
Sampling sites of *Apis cerana*. Samples from regions in blue were obtained from previously published data

We collected and resequenced samples from 10 regions of China and included previously published resequencing data from three regions (Table 1, Fig. 1). A total of 429.71 Gb of raw data were obtained from 100 samples. The total data volume after quality control was 418.97 Gb, the average alignment rate with the reference genome was 90.98%, and the average sequencing depth was 19.16 × . The average Q20 was 96.65% and the Q30 averaged 91.59%.

**Table 1:** Information on sampling sites

Collection site	Province	Sample code	Geographic coordinates	Climate type
Mangkang	Tibet	MK	29.48°N, 99.06°E	Plateau climate
Shennongjia	Hubei	SNJ	31.34°N, 110.55°E	Subtropical monsoon climate
Jinzhai	Anhui	JZ	31.39°N, 115.75°E	Subtropical monsoon climate
Qimen	Anhui	QM	29.70°N, 117.68°E	Subtropical monsoon climate
Suzhou	Jiangsu	SZ	31.13°N, 120.30°E	Subtropical monsoon climate
Guangzhou	Guangdong	GZ	23.47°N, 113.56°E	Subtropical monsoon climate
Wenchang	Hainan	WC	19.52°N, 110.59°E	Tropical monsoon climate
Jilin^a^	Jilin	JL	41.95–43.86°N, 125.6–129.76°E	Temperate monsoon climate
Mengyin^a^	Shandong	MY	35.65°N, 117.92°E	Warm temperate Monsoon climate
Zhangzhou^a^	Fujian	ZZ	24.51°N, 117.65°E	Subtropical monsoon climate

^a^samples were obtained from previously published data

### Population genetic structure analysis

**Fig. 2:**
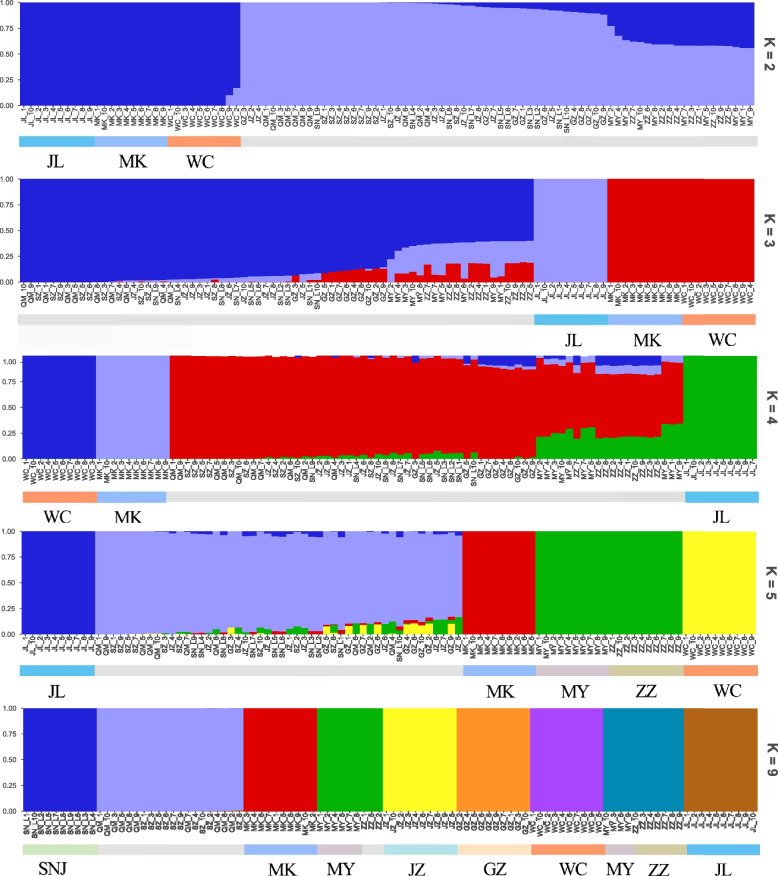
Analysis of population structure

**Fig. 3:**
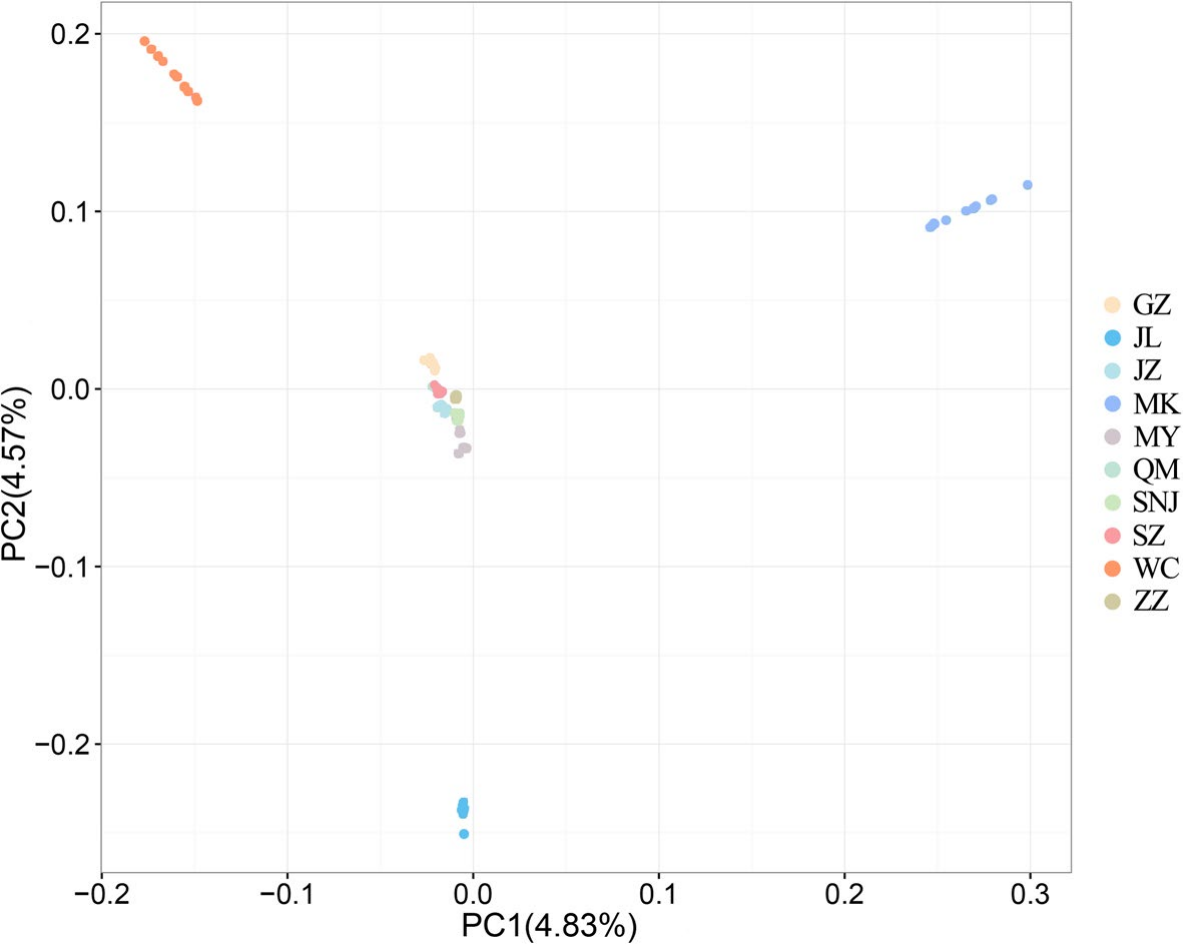
Principal component analysis of the 10 populations

**Fig. 4:**
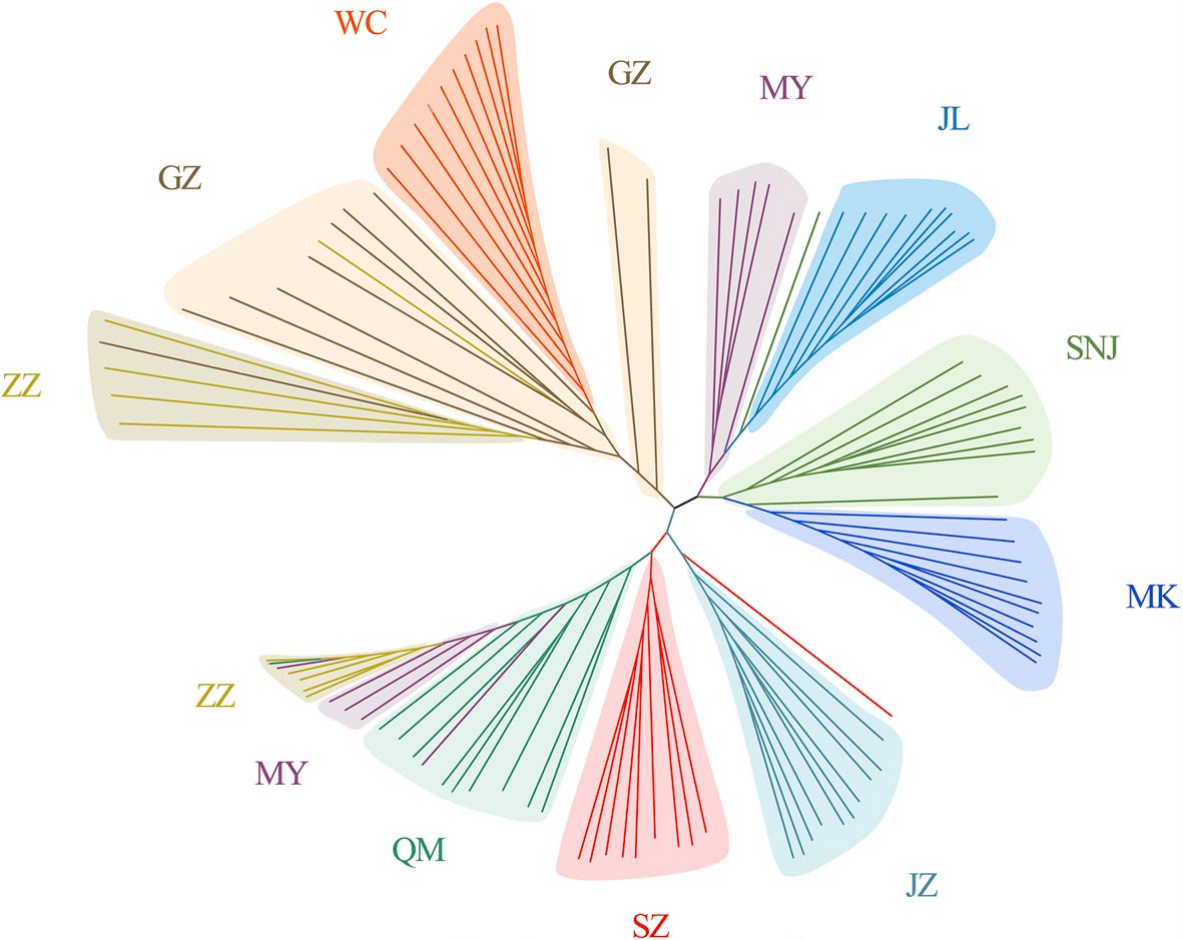
Phylogenetic tree of *Apis cerana*

The admixture model-based software (Admixture) was used to estimate the population structure. With K = 2, MK, JL, and WC formed an ancestral cluster, while the other populations showed different degrees of mixed lineage. As K increased from 3 to 5, MK, JL, and WC showed distinct lineages from the other populations, with ZZ and MY forming an ancestral cluster (Fig. 2). Simultaneously, we calculated the cross-validation error rate, which was the smallest when K = 4, of genetic structure analysis of 100 samples (Fig. S1). In particular, the SZ and QM groups remained indistinguishable when K increased from 4 to 9 (Fig. 2). The population subdivision pattern classified by principal component analysis (PCA) showed similar results. According to the first and second principal components, MK, JL, and WC were separated clearly (Fig. 3). The results of the group structure analysis and PCA of the 10 regional groups demonstrated that JL, MK, and WC could be separated from the remaining closely related and difficult-to-separate populations. The JL, MK, and WC groups were highly differentiated compared with the other groups. Based on the obtained SNP information, we used neighbor-joining methods to construct a phylogenetic tree (Fig. 4).

### Genetic differentiation and genetic diversity

To understand the genetic differentiation between the 10 groups, we calculated pairwise *F*_ST_ (Table 2) and genetic diversity parameters, including *Ho*, *He*, and *PIC* (Table 3). The maximum value of *F*_ST_ was 0.39 (between WC and JL), and the minimum value was less than 0.01 (a value close to 0, rounded to two decimal places is 0.00). These results also showed that JL, MK, and WC were in a state of high genetic differentiation (*F*_ST_ > 0.15), which is consistent with the analysis of population structure and PCA. JL displayed the highest degree of genetic differentiation (average *F*_ST_ = 0.28), followed by WC (average *F*_ST_ = 0.19) and MK (average *F*_ST_ = 0.16). The remaining regions were in a state of low genetic differentiation. The parameter calculation results for genetic diversity were greatest for SZ (*PIC* = 0.205) and GZ (*PIC* = 0.206) among the 10 populations. The genetic diversity of JL (*PIC* = 0.099), MK (*PIC* = 0.140), and WC (*PIC* = 0.148) was lower than that of other regions, indicating that bees in these three areas have been subjected to a higher intensity of natural selection.

**Table 2:** Pairwise *F*_ST_ distance between 10 populations of *Apis cerana*

Samples	GZ	JL	JZ	MK	MY	QM	SNJ	SZ	WC	ZZ
GZ	-									
JL	0.250	-								
JZ	0.010	0.250	-							
MK	0.110	0.370	0.120	-						
MY	0.040	0.270	0.030	0.150	-					
QM	0.000	0.250	0.000	0.120	0.030	-				
SNJ	0.010	0.240	0.010	0.120	0.030	0.010	-			
SZ	0.000	0.250	0.000	0.120	0.030	0.010	0.000	-		
WC	0.130	0.390	0.150	0.260	0.190	0.140	0.150	0.140	-	
ZZ	0.020	0.290	0.030	0.140	0.040	0.020	0.030	0.020	0.170	-

**Table 3:** Genetic diversity parameters of *Apis cerana*

Sample	*Ho*	*He*	*PIC*
JL	0.110	0.124	0.099
MK	0.250	0.176	0.140
WC	0.267	0.185	0.148
ZZ	0.162	0.180	0.150
MY	0.155	0.184	0.152
JZ	0.303	0.237	0.194
QM	0.333	0.248	0.203
SNJ	0.334	0.248	0.203
SZ	0.342	0.250	0.205
GZ	0.342	0.251	0.206
Mean	0.260	0.208	0.170

### Historical effective population sizes

**Fig. 5:**
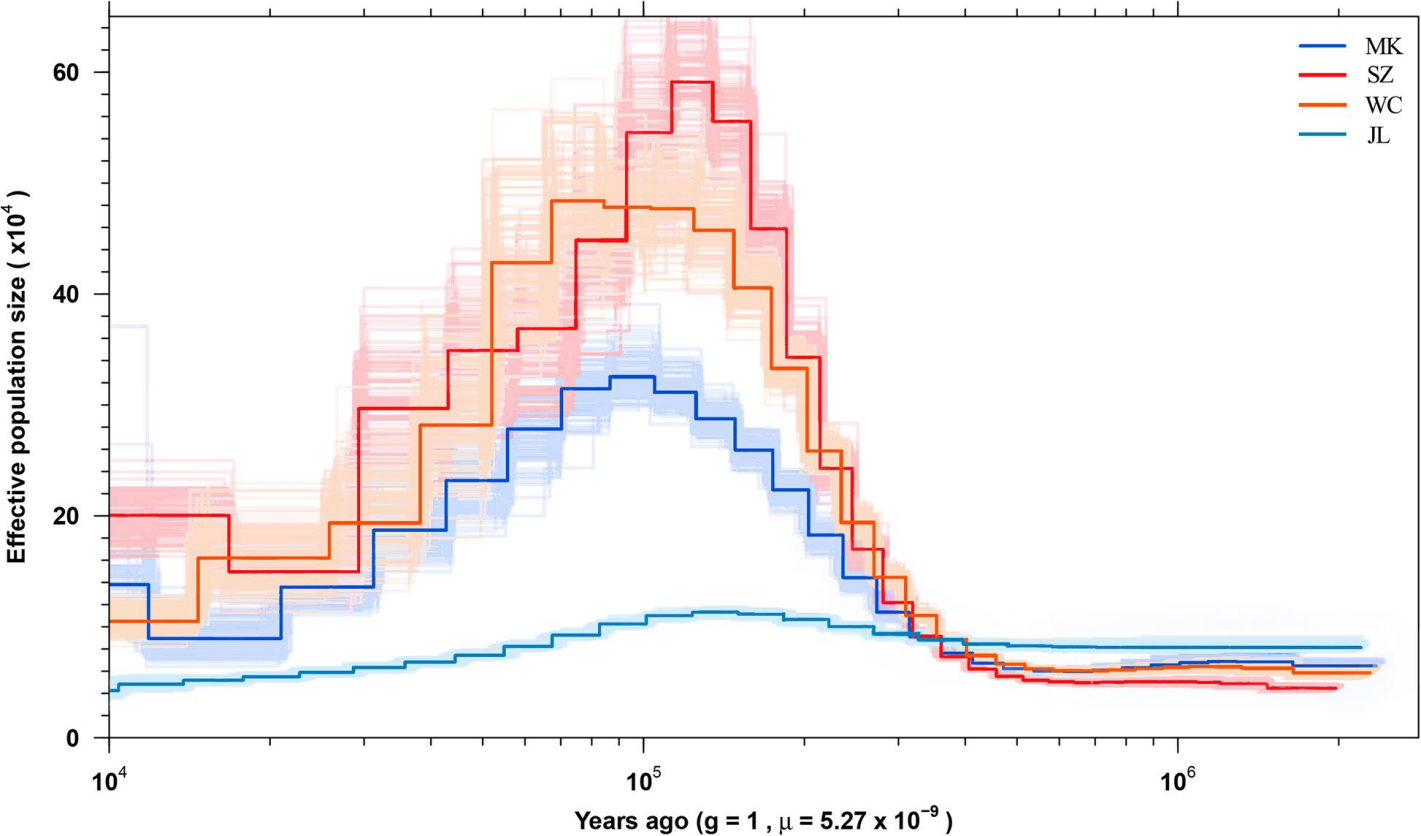
Estimation of historical effective population sizes

Based on the above population genetic structure analysis results, PCA, and genetic differentiation analysis, JL, MK, and WC have each independently become a subpopulation separated from the remaining populations. Moreover, we noticed that these three populations, in addition to a fourth group comprising the remaining seven populations, each occupy different regional climate types. Jilin is in the northeastern part of China and has a temperate continental monsoon climate; Mangkang is on the Qinghai-Tibet Plateau of China and has a typical plateau climate; Wenchang is on Hainan Island, China, with a unique tropical monsoon island-ridge climate; and the remaining populations are mostly in the subtropical monsoon climate region. The SZ group was selected as the representative subtropical monsoon climate region. To understand the important evolutionary events that occurred during the past adaptation of these four highly differentiated populations of *A. cerana*, we estimated their historical effective population sizes (Fig. 5). According to the estimated results, the four regions had nearly the same effective colony size during the period 80–200 ka years ago. About 70 ka years ago, the JL group began to show a change in population size that was different from that of the rest of the group. The population size of the three regions of MK, WC, and SZ in the middle and low latitudes showed an upward trend at this time, which seems to indicate that the population in these regions could begin to have continuous differentiation of population structure and mixed blood, resulting in an increase in population size, whereas the JL region did not undergo this process. Overall, the evolution of populations at high latitudes showed different trends than those at low and middle latitudes.

### Morphometric analysis

**Fig. 6:**
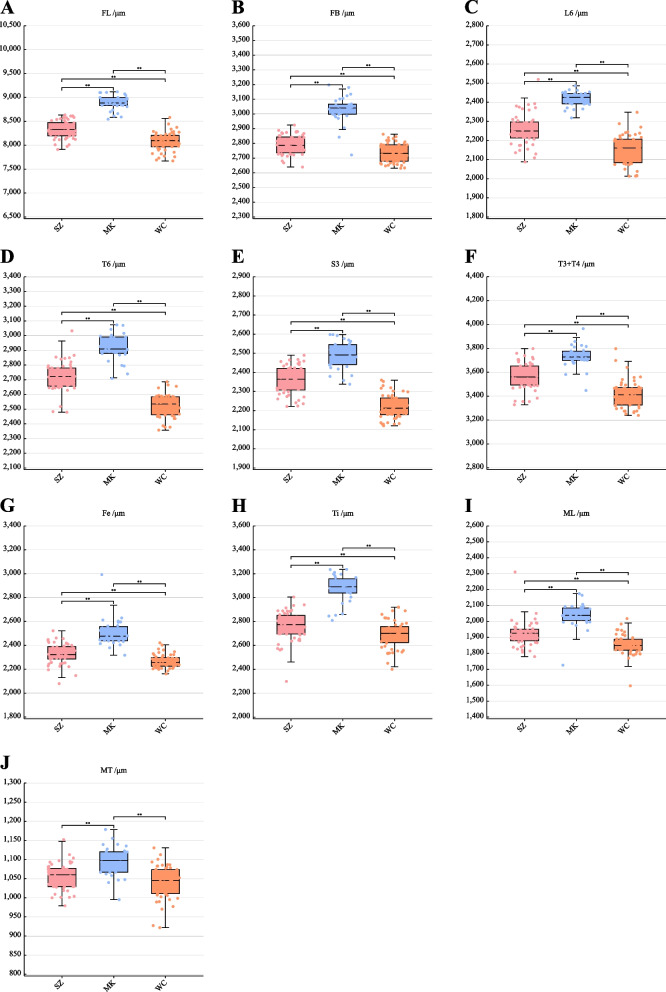
Boxplot of 10 morphological indicators of *Apis cerana* among the three geographical populations. **A** The right forewing length (FL). **B** The right forewing width (FB). **C** The sixth sternum length (L6). **D** The sixth sternum width (T6). **E** The third sternum length (S3). **F** The third tergum length and the fourth tergum length (T3 + T4). **G** The femur length (Fe). **H** The tibia length (Ti). **I** The basitarsus length (ML). **J** The basitarsus width (MT). Note: ** means significant difference at 0.01 level

We measured 10 morphological indicators related to body size on the samples collected from WC, MK, and SZ for morphometric analysis, including the right forewing length (FL), the right forewing width (FB), the sixth sternum length (L6), the sixth sternum width (T6), the third sternum length (S3), the third tergum length and the fourth tergum length (T3 + T4), the femur length (Fe), the tibia length (Ti), the basitarsus length (ML), and the basitarsus width (MT) (Fig. S2). The results of one-way ANOVA on the measured 10 indicators showed that, except for the insignificant difference between the MT of the SZ and WC groups (*P* > 0.05), all other indicators were significantly different among the three groups (*P* < 0.01) (Table 4 and Fig. 6). These results confirm that, in terms of body size, MK population is significantly larger than that of SZ, which in turn is significantly larger than that of WC.

**Table 4:** Summary morphological data of the three geographical populations

Items/μm	SZ	WC	MK
FL	8331.13 ± 26.53^a^	8074.91 ± 27.68^b^	8882.34 ± 28.50^c^
FB	2791.07 ± 9.08^a^	2735.01 ± 9.07^b^	3031.05 ± 16.88^c^
L6	2257.05 ± 11.71^a^	2153.16 ± 10.91^b^	2415.90 ± 7.09^c^
T6	2720.43 ± 13.67^a^	2523.80 ± 10.81^b^	2916.17 ± 16.17^c^
S3	2359.32 ± 10.33^a^	2221.33 ± 8.87^b^	2490.51 ± 13.15^c^
T3 + T4	3567.59 ± 15.91^a^	3415.16 ± 16.41^b^	3729.79 ± 16.99^c^
Fe	2329.92 ± 12.12^a^	2267.87 ± 7.95^b^	2504.20 ± 21.66^c^
Ti	2759.91 ± 17.55^a^	2691.96 ± 16.66^b^	3074.32 ± 21.23^c^
ML	1924.61 ± 11.36^a^	1853.14 ± 9.20^b^	2038.29 ± 14.37^c^
MT	1055.57 ± 4.84^a^	1040.64 ± 6.15^a^	1095.52 ± 7.14^b^

^a^^−^^c^different letters mean significant difference at 0.01 level

### Selective sweep analysis

To further study the adaptive radiation distribution, such as the difference in body size of *A. cerana* from different regions of China and the genome-level changes that occurred during the evolution of adaptation to the unique climate of each region under natural selection pressure, we estimated pairwise genetic differentiation (*F*_ST_) and differences in nucleotide diversity (π ratios) from the four different types of populations to identify key selective sweeps. For the 40 samples from JL, MK, WC, and SZ, we performed the linkage disequilibrium (LD) decay analysis. The LD analysis results showed that when r^2^ was half of the maximum value, the decay rate was MK > JL > WC > SZ (Fig. S3). This also indicates that the bees in MK, JL, and WC were affected by more intense selection pressure than those in SZ; therefore, we selected the SZ region with richer genetic diversity and less selection pressure as the reference population. The JL, MK, and WC populations were scanned and analyzed against the SZ reference population to identify the regions in the *A. cerana* genome that have been selected under natural selection pressure, thereby revealing the adaptive evolution of *A. cerana*.

**Fig. 7:**
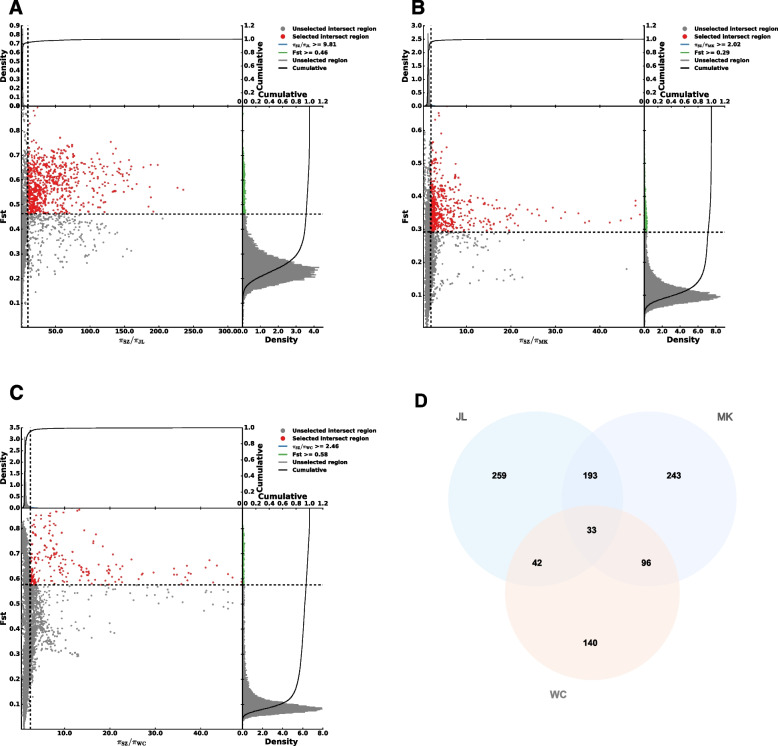
Selective sweep analysis against the reference SZ population. **A** Results of selective sweep analysis (SZ/JL). **B** Results of selective sweep analysis (SZ/MK). **C** Results of selective sweep analysis (SZ/WC). The selected intersect regions in red areas are at the top 5% level by *F*_ST_ and π ratios. **D** Venn diagram of selected genes

Selective sweep regions were chosen according to the intersection of two indices (*F*_ST_ and π ratios) with a threshold of the top 5% level. The results of the selection signal analysis of the highly differentiated populations under different climate types, with the SZ population (subtropical monsoon oceanic climate) as the reference population, identified 839 candidate regions involving 527 genes (Fig. 7A and Table S3) in the JL population (temperate monsoon climate). In the MK population (plateau climate), 589 candidate regions involving 565 genes were identified (Fig. 7B and Table S4). In the WC population (tropical monsoon island climate), 224 candidate regions involving 311 genes were identified (Fig. 7C and Table S5). In addition, 33 genes were identified in the selection signal analysis results for all three regions (Fig. 7D and Table S6). These 33 genes may play important roles in the adaptation of *A. cerana* to different climatic conditions in China. Gene Ontology (GO) and Kyoto Encyclopedia of Genes and Genomes (KEGG) pathway enrichment analyses were performed on these 33 candidate genes. The 33 shared genes were involved in 846 GO and 44 KEGG pathways, with 138 GO and 6 KEGG pathways significantly enriched at a threshold of *P* < 0.05 (Table S7 and Table S8). GO enrichment analysis revealed that the biological process with the most enriched genes was the single-organism process, the cellular component with the most enriched genes were cell and cell part, and the molecular function with the most enriched genes was binding (Fig. S4). The six signaling pathways were amino sugar and nucleotide sugar metabolism, peroxisome, RNA degradation, AMPK signaling pathway, ubiquitin-mediated proteolysis, and 2-oxocarboxylic acid metabolism. These results indicate that *A. cerana* responds to natural environmental stress primarily by regulating sugar and protein metabolism. In addition, among the top 20 enriched signaling pathways, we noticed some that may be related to the adaptation of honeybees to regional climates, such as the citrate cycle (TCA cycle) related to energy metabolism in the body, the thermogenesis pathway related to adaptation to changes in ambient temperature, and the Hedgehog signaling pathway involved in regulating gene expression (Fig. S5).

## Discussion

Our study results reveal an important link between climate type and the adaptive evolution of *A. cerana* in China. *A. cerana* with unique phenotypic variations from different climate regions (Jilin, Mangkang, and Wenchang) could have independently formed a subpopulation from the analyses of the population structure, genetic diversity, and genetic differentiation based on whole-genome resequencing data. Furthermore, we confirmed the significant differences in the body size of *A. cerana* by comparing morphological data. This evidence shows that the JL, MK, and WC populations were highly differentiated compared with other populations, which may be a consequence of different climate types and environmental selection pressures. This is in line with the findings of a study on the adaptive evolution of *A. cerana* in which isolated groups showed a high degree of differentiation and lower genetic diversity, whereas other groups were less differentiated and had higher genetic diversity [14]. In the present study, we found that the average *PIC* of the 10 populations was 0.17, and the *PIC* values of all populations were lower than 0.5, representing low polymorphism and low genetic diversity of *A. cerana*. Human activities are considered to be an important reason for the decrease in the genetic diversity of *A. cerana* in recent years. Human activities led to the reduction of the habitat area of *A. cerana*, which directly led to the reduction of wild resources of *A. cerana*. A large reduction in the number of wild bees leads to a decrease in the effective population, which may directly lead to a decrease in the genetic diversity of *A. cerana*. In addition, the introduction of *A. cerana* from other regions also affected the original genetic structure and genetic diversity of local populations. A rich genetic diversity can enhance the adaptability of species to their environment, more stably maintain population balance [28, 29], and improve production performance [30]. Therefore, it is imperative to protect and rationally utilize the genetic resources of *A. cerana*.

It is also worth noting that the geographic latitude factor had a high degree of influence on the biodiversity of biological groups. This is supported by the estimations of the effective population size. The different change patterns of the effective population size of *A. cerana* at high and low latitudes also reflect the different impacts of sudden climate change on populations at different latitudes. Our study shows that this discrepancy first originated around 70 to 80 ka years ago. Since the last glacial period of the Quaternary Ice Age [31, 32], different degrees of cooling have occurred throughout China, where the temporal and spatial differences in the cooling rates are remarkable. A significant feature is that cooling is greater in winter than in summer and at high latitudes than at low latitudes. This widespread cooling may have prompted the migration and fusion of high-latitude regions with low-latitude regions. During this process, species at high latitudes could be subjected to stronger natural selection than those at low latitudes.

The main driving forces in the process of species evolution include selection and genetic drift. The joint action of multiple forces may not only make the mutant increase its frequency faster, but also may be that genetic drift slows down the speed of selection, or even reduces the frequency of the mutant. The intensity of genetic drift depends on the effective population size. The larger the effective population size, the weaker the genetic drift; the smaller the effective population size, the higher the probability of genetic drift. According to our estimation of the effective population size, the effective population size of *A. cerana* in Jilin is lower than that of other populations. This means that *A. cerana* in Jilin may have experienced serious bottleneck events, and the evolution is more affected by genetic drift. This is consistent with the previously reported situation that human capture has greatly changed the population dynamics of *A. cerana* in the area around JL in recent decades [22, 25]. Therefore, in order to reduce the false positives caused by the influence of genetic drift and other factors on the SNP scan, we increase the sensitivity of the selection signal by calculating the *F*_ST_ between populations in the sliding window.

In this study, we mainly focus on the relationship between genetic variations and climatic conditions. Hence, we pay attention to the factors like latitude, longitude, and altitude related to climate conditions. It is worth noting that in the process of adaptive evolution, especially in the case of short generation interval of bees, the impact of artificial domestication cannot be ignored. Both the changes in genome structure characteristics caused by artificial selection and natural selection are called selection signals. For these selection signals, it is difficult to distinguish whether their source is artificial selection or natural selection. Therefore, when using the selective sweep analysis to analyze the evolution of species, it is necessary to select appropriate research objects according to the direction of focus. We selected *A. cerana* as the research object rather than *Apis mellifera* to study climatic adaptation. Because the domestication history of *A. cerana* is shorter than that of *A. mellifera*, and the degree of domestication is weaker than that of *A. mellifera*. In addition, during the sampling process, the samples we collected were *A. cerana* living in different geographical environments in the wild or semi-wild state. These samples are relatively less affected by artificial domestication. We believe that such samples are suitable for studying climatic adaptation during the adaptive evolution of *A. cerana*, and the analysis results based on these samples are representative.

Long-term natural selection often causes changes in the allele frequencies of selected loci and their linked loci. Upon analyzing these traces of natural selection in the genome, we can better understand some of the important events in evolution. We used selective sweep analysis to determine the relationship between climate type and the adaptive evolution of *A. cerana*. Positive selection will lead to an increase in the frequency of the dominant allele at the locus, which in turn will lead to a decrease in the genetic polymorphism of the selected locus. Therefore, for the studied colonies of *A. cerana* under the four climatic types, we set the SZ population as the reference, based on the calculated population polymorphism (SZ > WC > MK > JL). An abundance of polymorphisms means that the selection pressure received is less; hence using the SZ population with less selection pressure as the background facilitates the detection of traces of selection in the remaining populations. In addition, we use different selective signal methods (*F*_ST_ and π ratios) for selective signal detection. The adoption of an overlapping detection strategy can further avoid the occurrence of false positives through mutual verification between methods to a certain extent. It is undeniable that the results obtained after these pairwise comparisons are relatively rich, but not all signals are related to adaptation to the climate environment and body size; therefore, we pay more attention to the signals detected in all three selective sweep analyses. These signals will help gain insights into the climatic adaptation mechanisms of *A. cerana*. According to the results of GO and KEGG pathway analyses, we speculate that the adaptive evolution of *A. cerana* may be preferentially reflected in its response to the abundance of food resources and changes in temperature. *A. cerana* actively regulates its metabolism to cope with food shortages in harsh environments and maintain its body temperature. This also indicates that *A. cerana* energy utilization patterns may differ in different climate types.

Based on our findings, we speculate that the selection of RAPTOR could help *A. cerana* adapt to the complex climatic environment in China. A total of 33 candidate genes related to climatic adaptation were screened using selective elimination analysis. Upon annotation results combination with existing research reports, we identified RAPTOR as a candidate gene related to body size in climate adaptation. RAPTOR is the intersection of the AMPK and mTOR signaling pathways. The evidence to date suggests that the mTOR signaling pathway is involved in the development of *A. mellifera* [33–36]. Studies have shown that the mTOR signaling pathway plays a key role in the development of the queen bee [37–41], and the knockdown of the TOR encoding gene can affect the fate of the queen [37]. Under nutrient deprivation, AMPK, an important kinase that regulates energy homeostasis, directly phosphorylates RAPTOR to inhibit the mTORC1, thereby inhibiting cell growth [42]. The mTORC1 participates in glucose metabolism indirectly in response to insulin secreted by glucose entering the blood or is directly activated by amino acids [43, 44]. The mTOR pathway is also involved in regulating autophagy. When insufficient nutrient supply causes insufficient mTORC1 activity, mTORC1 initiates the inhibition of autophagy and activates lysosomes to degrade relatively uncritical proteins and organelles to provide material and energy to maintain the basic survival needs of cells [45, 46]. Bees absorb nutrients (glucose and amino acids) by feeding on plant pollen and nectar [23]. Native plants are affected by local climatic conditions, exhibiting shorter flowering periods and lower abundance in colder regions than in warmer regions [26, 47]. As a result, the degree of difficulty in finding food and absorbing nutrients for *A. cerana* in different regions is inconsistent. The selection of RAPTOR at the genome level could be in response to changes in climate and food sources. Early studies also reported the functions of the RAPTOR and TOR pathways in *Drosophila* [43, 46, 48, 49]. Decreased TOR activity inhibits ecdysone release and leads to prolonged development time and increased body size, whereas activating TOR can reverse stunting caused by nutrient restriction [50]. In addition, RAPTOR was upregulated in a study of cold resistance in *Drosophila* [51]. A recent study on thermal and oxygen flight sensitivity in *Drosophila* showed that downregulation of TOR activity produces smaller flies with smaller wing epidermal cells, and flies with small cells can maintain superior performance in metabolically demanding activities, such as flying under hypoxic conditions [52]. Whether *A. cerana* has adopted the same strategy as other insects to combat different climate types requires further investigation.

In summary, our findings suggest that climate type, geographic location, and food resources are all related to the adaptive radiation distribution and unique geographic phenotypes of *A. cerana*. It is highly probable that food and nutrition deficits caused by diverse climates are the predominant driving forces for unique geographic phenotypes. Further research is necessary to verify the molecular mechanism between RAPTOR and the body size.

## Conclusions

Our study shows that the genetic structure and genetic differentiation of *A. cerana* are related to the distribution of climate and environment in China and are strongly affected by latitude. A key gene, RAPTOR, plays an important role in this process. The selection of RAPTOR at the genomic level helps *A. cerana* respond to nutritional problems caused by harsh environments by modulating TOR activity to alter body size, which partly explains the difference in body size among *A. cerana* distributed in China. These results help us understand the genetic basis of the climatic adaptation of *A. cerana* and provide a reference for the protection and utilization of germplasm resources and future genetic improvement.

## Methods

### Sample collection

A total of 100 honeybee samples were studied in this experiment, 70 of which were collected from Tibet, Hubei, Anhui, Jiangsu, Guangdong, and Hainan provinces in China. The collection sites for each sample were centered in Suzhou, Jiangsu, and were located at similar latitudes and different longitudes or similar longitudes and different latitudes. In addition, 30 samples from sites with similar longitude and different latitude as Suzhou were included for comprehensive data analysis [14, 18]. Ten colonies of 3–5 bees were collected from each sampling point. The collected bee samples included wild and semi-wild local bees. Samples were placed in 75% alcohol and then stored at -20 °C for future use.

### Whole-genome sequencing, quality control, and clean reads mapping

Total genomic DNA was extracted from samples, and at least 3 µg genomic DNA was used to construct paired-end libraries with an insert size of 500 bp using a Paired-End DNA Sample Prep kit (Illumina Inc., San Diego, CA, USA). These libraries were sequenced using the HiSeq X10 NGS platform (Illumina Inc., San Diego, CA, USA). Raw reads were processed to obtain high-quality clean reads according to two stringent filtering standards: 1) removing reads containing > 50% of low-quality bases (Q < 20) or > 10% unidentified nucleotides (Ns); and 2) removing reads aligned to the barcode adapter. The Burrows-Wheeler Aligner (BWA) was used to align the clean reads against the reference genome (GCA_011100585.1) with the settings ‘mem 4 -k 32 -M’ [53]. Duplicates were marked using Picard 2.18.7 (http://broadinstitute.github.io/picard/).

### Variant identification and annotation

Variant identification was performed using the Genome Analysis Toolkit (GATK) [54]. SNPs were filtered using GATK Variant Filtration with proper standards (-Window 4, -filter "QD < 2.0 || FS > 60.0 || MQ < 40.0", -G_filter "GQ < 20"). SNPs were filtered according to two stringent filtering standards: 1) missing ratio < 20%; and 2) minor allele frequency (MAF) > 5%. The ANNOVAR software [55] was used to annotate SNPs.

### Principal component analysis (PCA), population structure analysis, and phylogenetic analysis

The admixture model-based software, Admixture V1.3.0 [56], was used to estimate the population structure. The tested K was set from 1 to 9, and the optimal K was determined based on the lowest cross-validation error. The population subdivision pattern was preliminarily classified using PCA in the GCTA software [57]. We constructed a phylogenetic tree using the neighbor-joining method with TreeBeST software [58]. The bootstrapped confidence interval was based on 1000 replicates.

### Genetic diversity and *FST* statistics

*Ho* and *He* for each group or population were calculated using Plink [59]. *Ne* and *PIC* were calculated using the Perl script. The pairwise *F*_ST_ matrix was calculated using Genepop software [60].

### Historical effective population sizes

SMC +  + 1.13.1 [61] was used to estimate the changes in *Ne* over the past one million years (Ma). The mutation rate was set to 5.27 × 10^−9^ per base pair per generation, following a divergence estimate of 7 Ma between *A. mellifera* and *A. cerana* [62]. The generation time was assumed to be one year. The polarization error was set to 0.5.

### Morphometric analysis

A total of 130 worker bees (30 from MK, 50 from WC, and 50 from SZ) were used for morphometric measurements. The bees were dissected to make samples of each tissue, observed and photographed under a stereo microscope, and measured by a measurement system (M-Shot Image Analysis System V1.1.4). The measure of each sample in parallel was repeated 3 times and the mean value was taken.

### Linkage disequilibrium (LD)

To evaluate the LD pattern, we estimated the squared allele frequency correlation (r^2^) using Haploview 4.2 [63]. LD decay graphs were plotted using the R script.

### Selective sweep analysis

For the 40 samples from JL, MK, WC, and SZ, we performed selective sweep analysis, with SZ as the reference population. We estimated pairwise genetic differentiation (*F*_ST_) [64] and differences in nucleotide diversity (π ln-ratio) [65] from the four different populations to identify key selective regions using the PopGenome software [66] with the sliding window approach. We set the window size to 100 kb and the step size to 10 kb. Selective sweep regions were selected according to the interception of two indices (*F*_ST_ and π ratios), with a threshold of the top 5% level. All related graphs were drawn using R scripts. Candidate genes within sweep regions were extracted for GO and KEGG enrichment analysis.

## Supplementary Information

**Additional file 1:** **Figure S1.** The cross-validation error rate of genetic structure analysis of 100 samples. **Figure S2.** The diagram of 10 morphological indicators of *A.cerana*. (A) The right forewing length (FL) and width (FB). (B) The sixth sternum length (L6) and width (T6). (C) The third sternumlength (S3). (D) The third tergum length (T3). (E) The fourth tergum length (T4). (F) The femur length (Fe), the tibia length (Ti), the basitarsus length (ML), and the basitarsus width (MT). **Figure S3.** Analysis of linkage disequilibrium. **Figure S4.** GO classification of candidate genes. **Figure S5.** The top 20 enriched KEGG pathways.

**Additional file 2:** **Table S1.** Sequencing data production and quality statistics of 100 samples. **Table S2.** Summary of sequencing data depth, coverage, and mapping rate of 10 sampling sites. **Table S3.** 527 selected genes of *A. cerana* in Jilin. **Table S4.** 565 selected genes of *A. cerana* in Mangkang. **Table S5.** 311 selected genes of *A. cerana* in Wenchang. **Table S6.** 33 common selected genes of the three geographical populations. **Table S7.** GO enrichment of the 33 common selected genes of *A. cerana* (*P *< 0.05). **Table S8.** KEGG enrichment of the 33 common selected genes of *A. cerana* (*P *<0.05).

## Data Availability

Sequencing data of 70 samples in this study have been submitted to the NCBI Short Read Archive (SRA) under the BioProject accession number PRJNA876655. The morphometry data and other data are included in this paper.

## References

[1] Hallmann CA, Sorg M, Jongejans E, Siepel H, Hofland N, Schwan H, Stenmans W, Muller A, Sumser H, Horren T, Goulson D, de Kroon H (2017). More than 75 percent decline over 27 years in total flying insect biomass in protected areas. PLoS ONE.

[2] Soroye P, Newbold T, Kerr J (2020). Climate change contributes to widespread declines among bumble bees across continents. Science.

[3] Salcido DM, Forister ML, Lopez HG, Dyer LA (2020). Loss of dominant caterpillar genera in a protected tropical forest. Sci Rep.

[4] Harris JE, Rodenhouse NL, Holmes RT (2019). Decline in beetle abundance and diversity in an intact temperate forest linked to climate warming. Biol Conserv.

[5] van der Zee R, Pisa L, Andonov S, Brodschneider R, Charriere JD, Chlebo R, Coffey MF, Crailsheim K, Dahle B, Gajda A, Gray A, Drazic MM, Higes M, Kauko L, Kence A, Kence M, Kezic N, Kiprijanovska H, Kralj J, Kristiansen P, Hernandez RM, Mutinelli F, Nguyen BK, Otten C, Ozkirim A, Pernal SF, Peterson M, Ramsay G, Santrac V, Soroker V, Topolska G, Uzunov A, Vejsnaes F, Wei S, Wilkins S (2012). Managed honeybee colony losses in Canada, China, Europe, Israel and Turkey, for the winters of 2008–9 and 2009–10. J Apic Res.

[6] Scheffers BR, De Meester L, Bridge TCL, Hoffmann AA, Pandolfi JM, Corlett RT, Butchart SHM, Pearce-Kelly P, Kovacs KM, Dudgeon D, Pacifici M, Rondinini C, Foden WB, Martin TG, Mora C, Bickford D, Watson JEM (2016). The broad footprint of climate change from genes to biomes to people. Science.

[7] Halsch CA, Shapiro AM, Fordyce JA, Nice CC, Thorne JH, Waetjen DP, Forister ML (2021). Insects and recent climate change. Proc Natl Acad Sci U S A.

[8] Warren R, Price J, Graham E, Forstenhaeusler N, VanDerWal J (2018). The projected effect on insects, vertebrates, and plants of limiting global warming to 1.5 degrees C rather than 2 degrees C. Science.

[9] Roman-Palacios C, Wiens JJ (2020). Recent responses to climate change reveal the drivers of species extinction and survival. Proc Natl Acad Sci U S A.

[10] Urban MC (2015). Accelerating extinction risk from climate change. Science.

[11] Garcia RA, Cabeza M, Rahbek C, Araujo MB (2014). Multiple Dimensions of Climate Change and Their Implications for Biodiversity. Science.

[12] Boggs CL (2016). The fingerprints of global climate change on insect populations. Curr Opin Insect Sci.

[13] Hoffmann AA, Sgro CM (2011). Climate change and evolutionary adaptation. Nature.

[14] Chen C, Wang HH, Liu ZG, Chen X, Tang J, Meng FM, Shi W (2018). Population Genomics Provide Insights into the Evolution and Adaptation of the Eastern HoneyBee (Apis cerana). Mol Biol Evol.

[15] Shi P, Zhou J, Song H, Wu Y, Lan L, Tang X, Ma Z, Vossbrinck CR, Vossbrinck B, Zhou Z, Xu J (2020). Genomic analysis of Asian honeybee populations in China reveals evolutionary relationships and adaptation to abiotic stress. Ecol Evol.

[16] Ilyasov RA, Park J, Takahashi J, Kwon HW (2018). Phylogenetic Uniqueness of Honeybee from the Korean Peninsula Inferred from The Mitochondrial, Nuclear, and Morphological Data. J Apic Sci..

[17] Ilyasov RA, Youn HG, Lee ML, Kim KW, Proshchalykin MY, Lelej AS, Takahashi J, Kwon HW (2019). Phylogenetic Relationships of Russian Far-East Apis Cerana with Other North Asian Populations. J Apic Sci.

[18] Ji Y, Li X, Ji T, Tang J, Qiu L, Hu J, Dong J, Luo S, Liu S, Frandsen PB, Zhou X, Parey SH, Li L, Niu Q, Zhou X (2020). Gene reuse facilitates rapid radiation and independent adaptation to diverse habitats in the Asian honeybee. Sci Adv.

[19] Wallberg A, Schoning C, Webster MT, Hasselmann M (2017). Two extended haplotype blocks are associated with adaptation to high altitude habitats in East African honeybees. PLoS Genet.

[20] Wallberg A, Pirk CW, Allsopp MH, Webster MT (2016). Identification of Multiple Loci Associated with Social Parasitism in Honeybees. PLoS Genet.

[21] Chen C, Liu ZG, Pan Q, Chen X, Wang HH, Guo HK, Liu SD, Lu HF, Tian SL, Li RQ, Shi W (2016). Genomic Analyses Reveal Demographic History and Temperate Adaptation of the Newly Discovered HoneyBee Subspecies Apis mellifera sinisxinyuan n. ssp. Mol Biol Evol.

[22] Park D, Jung JW, Choi BS, Jayakodi M, Lee J, Lim J, Yu Y, Choi YS, Lee ML, Park Y, Choi IY, Yang TJ, Edwards OR, Nah G, Kwon HW (2015). Uncovering the novel characteristics of Asian honeybee, Apis cerana, by whole genome sequencing. BMC Genomics.

[23] Diao QY, Sun LX, Zheng HJ, Zeng ZJ, Wang SY, Xu SF, Zheng HQ, Chen YP, Shi YY, Wang YZ, Meng F, Sang QL, Cao LF, Liu F, Zhu YQ, Li WF, Li ZG, Dai CJ, Yang MJ, Chen SL, Chen RS, Zhang SW, Evans JD, Huang Q, Liu J, Hu FL, Su SK, Wu J (2018). Genomic and transcriptomic analysis of the Asian honeybee Apis cerana provides novel insights into honeybee biology. Sci Rep.

[24] Wang ZL, Zhu YQ, Yang Q, Yan WY, Zheng HJ, Zeng ZJ (2020). A Chromosome-Scale Assembly of the Asian Honeybee Apis cerana Genome. Front Genet.

[25] Liu NN, Liu HM, Ju Y, Li XA, Li Y, Wang TJ, He JM, Niu QS, Xing XM (2022). Geometric morphology and population genomics provide insights into the adaptive evolution of Apis cerana in Changbai Mountain. BMC Genomics.

[26] Lan L, Shi P, Song H, Tang X, Zhou J, Yang J, Yang M, Xu J (2021). De Novo Genome Assembly of Chinese Plateau Honeybee Unravels Intraspecies Genetic Diversity in the Eastern Honeybee, Apis cerana. Insects.

[27] Xu K, Niu Q, Zhao H, Du Y, Jiang Y (2017). Transcriptomic analysis to uncover genes affecting cold resistance in the Chinese honeybee (Apis cerana cerana). PLoS ONE.

[28] Oldroyd BP, Fewell JH (2007). Genetic diversity promotes homeostasis in insect colonies. Trends Ecol Evol.

[29] Jones JC, Myerscough MR, Graham S, Oldroyd BP (2004). Honeybee nest thermoregulation: diversity promotes stability. Science.

[30] Mattila HR, Seeley TD (2007). Genetic diversity in honeybee colonies enhances productivity and fitness. Science.

[31] Batchelor CL, Margold M, Krapp M, Murton D, Dalton AS, Gibbard PL, Stokes CR, Murton JB, Manica A (2019). The configuration of Northern Hemisphere ice sheets through the Quaternary. Nat Commun.

[32] Clark PU, Dyke AS, Shakun JD, Carlson AE, Clark J, Wohlfarth B, Mitrovica JX, Hostetler SW, McCabe AM (2009). The Last Glacial Maximum. Science.

[33] Ronai I, Vergoz V, Oldroyd BP (2016). The Mechanistic, Genetic, and Evolutionary Basis of Worker Sterility in the Social Hymenoptera. Adv Study Behav.

[34] Cardoen D, Wenseleers T, Ernst UR, Danneels EL, Laget D, De Graaf DC, Schoofs L, Verleyen P (2011). Genome-wide analysis of alternative reproductive phenotypes in honeybee workers. Mol Ecol.

[35] Chen H, Wu GA, Zhou H, Dai XY, Steeghs NWF, Dong XL, Zheng L, Zhai YF (2021). Hormonal Regulation of Reproductive Diapause That Occurs in the Year-Round Mass Rearing of Bombus terrestris Queens. J Proteome Res.

[36] Corby-Harris V, Snyder L, Meador C (2019). Fat body lipolysis connects poor nutrition to hypopharyngeal gland degradation in Apis mellifera. J Insect Physiol.

[37] Patel A, Fondrk MK, Kaftanoglu O, Emore C, Hunt G, Frederick K, Amdam GV (2007). The Making of a Queen: TOR Pathway Is a Key Player in Diphenic Caste Development. PLoS ONE.

[38] Mutti NS, Dolezal AG, Wolschin F, Mutti JS, Gill KS, Amdam GV (2011). IRS and TOR nutrient-signaling pathways act via juvenile hormone to influence honeybee caste fate. J Exp Biol.

[39] Wheeler DE, Buck NA, Evans JD (2014). Expression of insulin/insulin-like signalling and TOR pathway genes in honeybee caste determination. Insect Mol Biol.

[40] Corona M, Libbrecht R, Wheeler DE (2016). Molecular mechanisms of phenotypic plasticity in social insects. Curr Opin Insect Sci.

[41] Chen X, Hu Y, Zheng HQ, Cao LF, Niu DF, Yu DL, Sun YQ, Hu SN, Hu FL (2012). Transcriptome comparison between honeybee queen- and worker-destined larvae. Insect Biochem Mol Biol.

[42] Mihaylova MM, Shaw RJ (2011). The AMPK signalling pathway coordinates cell growth, autophagy and metabolism. Nat Cell Biol.

[43] Geminard C, Rulifson EJ, Leopold P (2009). Remote Control of Insulin Secretion by Fat Cells in Drosophila. Cell Metab.

[44] Duran A, Amanchy R, Linares JF, Joshi J, Abu-Baker S, Porollo A, Hansen M, Moscat J, Diaz-Meco MT (2011). p62 is a key regulator of nutrient sensing in the mTORC1 pathway. Mol Cell.

[45] Wei Y, Lilly MA (2014). The TORC1 inhibitors Nprl2 and Nprl3 mediate an adaptive response to amino-acid starvation in Drosophila. Cell Death Differ.

[46] Takats S, Varga A, Pircs K, Juhasz G (2015). Loss of Drosophila Vps16A enhances autophagosome formation through reduced Tor activity. Autophagy.

[47] Cleland EE, Chuine I, Menzel A, Mooney HA, Schwartz MD (2007). Shifting plant phenology in response to global change. Trends Ecol Evol.

[48] Guertin DA, Guntur KVP, Bell GW, Thoreen CC, Sabatini DM (2006). Functional Genomics identifies TOR-regulated genes that control growth and division. Curr Biol.

[49] Lee G, Chung J (2007). Discrete functions of rictor and raptor in cell growth regulation in Drosophila. Biochem Bioph Res Co.

[50] Layalle S, Arquier N, Leopold P (2008). The TOR pathway couples nutrition and developmental timing in Drosophila. Dev Cell.

[51] Telonis-Scott M, Hallas R, McKechnie SW, Wee CW, Hoffmann AA (2009). Selection for cold resistance alters gene transcript levels in Drosophila melanogaster. J Insect Physiol.

[52] Szlachcic E, Czarnoleski M (2021). Thermal and Oxygen Flight Sensitivity in Ageing Drosophila melanogaster Flies: Links to Rapamycin-Induced Cell Size Changes. Biology.

[53] Li H, Durbin R (2009). Fast and accurate short read alignment with Burrows-Wheeler transform. Bioinformatics.

[54] McKenna A, Hanna M, Banks E, Sivachenko A, Cibulskis K, Kernytsky A, Garimella K, Altshuler D, Gabriel S, Daly M, DePristo MA (2010). The Genome Analysis Toolkit: a MapReduce framework for analyzing next-generation DNA sequencing data. Genome Res.

[55] Wang K, Li M, Hakonarson H (2010). ANNOVAR: functional annotation of genetic variants from high-throughput sequencing data. Nucleic Acids Res.

[56] Alexander DH, Novembre J, Lange K (2009). Fast model-based estimation of ancestry in unrelated individuals. Genome Res.

[57] Yang J, Lee SH, Goddard ME, Visscher PM (2011). GCTA: a tool for genome-wide complex trait analysis. Am J Hum Genet.

[58] Vilella AJ, Severin J, Ureta-Vidal A, Heng L, Durbin R, Birney E (2009). EnsemblCompara GeneTrees: Complete, duplication-aware phylogenetic trees in vertebrates. Genome Res.

[59] Purcell S, Neale B, Todd-Brown K, Thomas L, Ferreira MA, Bender D, Maller J, Sklar P, de Bakker PI, Daly MJ, Sham PC (2007). PLINK: a tool set for whole-genome association and population-based linkage analyses. Am J Hum Genet.

[60] Diniz JAF, Soares TN, Lima JS, Dobrovolski R, Landeiro VL, Telles MPD, Rangel TF, Bini LM (2013). Mantel test in population genetics. Genet Mol Biol.

[61] Terhorst J, Kamm JA, Song YS (2017). Robust and scalable inference of population history from hundreds of unphased whole genomes. Nat Genet.

[62] Wallberg A, Han F, Wellhagen G, Dahle B, Kawata M, Haddad N, Simoes ZLP, Allsopp MH, Kandemir I, De la Rua P, Pirk CW, Webster MT (2014). A worldwide survey of genome sequence variation provides insight into the evolutionary history of the honeybee Apis mellifera. Nat Genet.

[63] Barrett JC, Fry B, Maller J, Daly MJ (2005). Haploview: analysis and visualization of LD and haplotype maps. Bioinformatics.

[64] Hudson RR, Slatkin M, Maddison WP (1992). Estimation of levels of gene flow from DNA sequence data. Genetics.

[65] Lin T, Zhu GT, Zhang JH, Xu XY, Yu QH, Zheng Z, Zhang ZH, Lun YY, Li S, Wang XX, Huang ZJ, Li JM, Zhang CZ, Wang TT, Zhang YY, Wang AX, Zhang YC, Lin K, Li CY, Xiong GS, Xue YB, Mazzucato A, Causse M, Fei ZJ, Giovannoni JJ, Chetelat RT, Zamir D, Stadler T, Li JF, Ye ZB, Du YC, Huang SW (2014). Genomic analyses provide insights into the history of tomato breeding. Nat Genet.

[66] Pfeifer B, Wittelsburger U, Ramos-Onsins SE, Lercher MJ (2014). PopGenome: An Efficient Swiss Army Knife for Population Genomic Analyses in R. Mol Biol Evol.

