# Supplementary material for: A key gene for the climatic adaptation of Apis cerana populations in China according to selective sweep analysis
Source: BMC Genomics. 2023 Mar 6;24:100. doi: 10.1186/s12864-023-09167-x (PMC9987060; doi:10.1186/s12864-023-09167-x)
Supplement: Supplementary file 1 — Additional file 1: Figure S1. The cross-validation error rate of genetic structure analysis of 100 samples. Figure S2. The diagram of 10 morphological indicators of A.cerana. (A) The right forewing length (FL) and width (FB). (B) The sixth sternum length (L6) and width (T6). (C) The third sternumlength (S3). (D) The third tergum length (T3). (E) The fourth tergum length (T4). (F) The femur length (Fe), the tibia length (Ti), the basitarsus length (ML), and the basitarsus width (MT). Figure S3. Analysis of linkage disequilibrium. Figure S4. GO classification of candidate genes. Figure S5. The top 20 enriched KEGG pathways. [file 12864_2023_9167_MOESM1_ESM.docx]

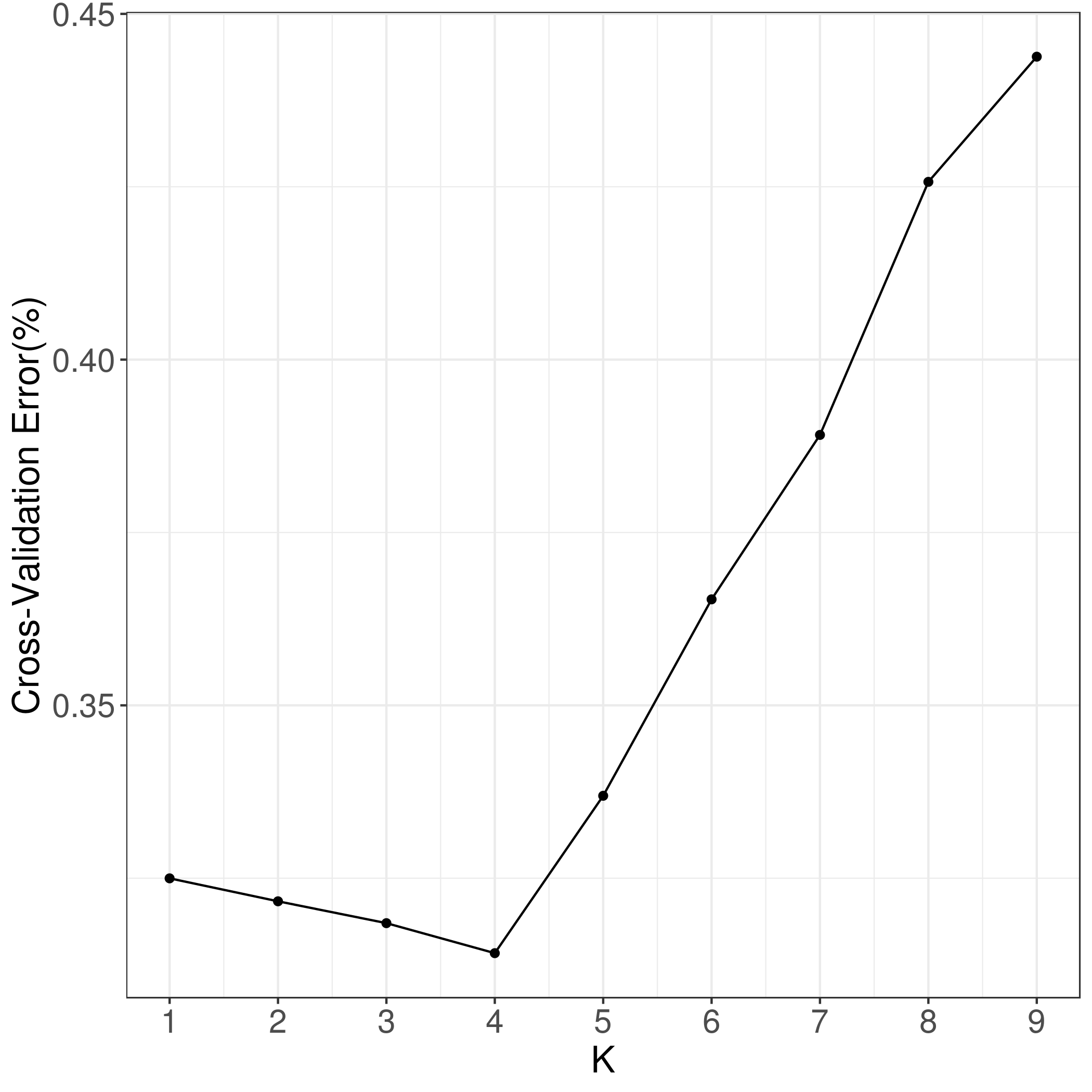


Fig. S1. The cross-validation error rate of genetic structure analysis of 100 samples


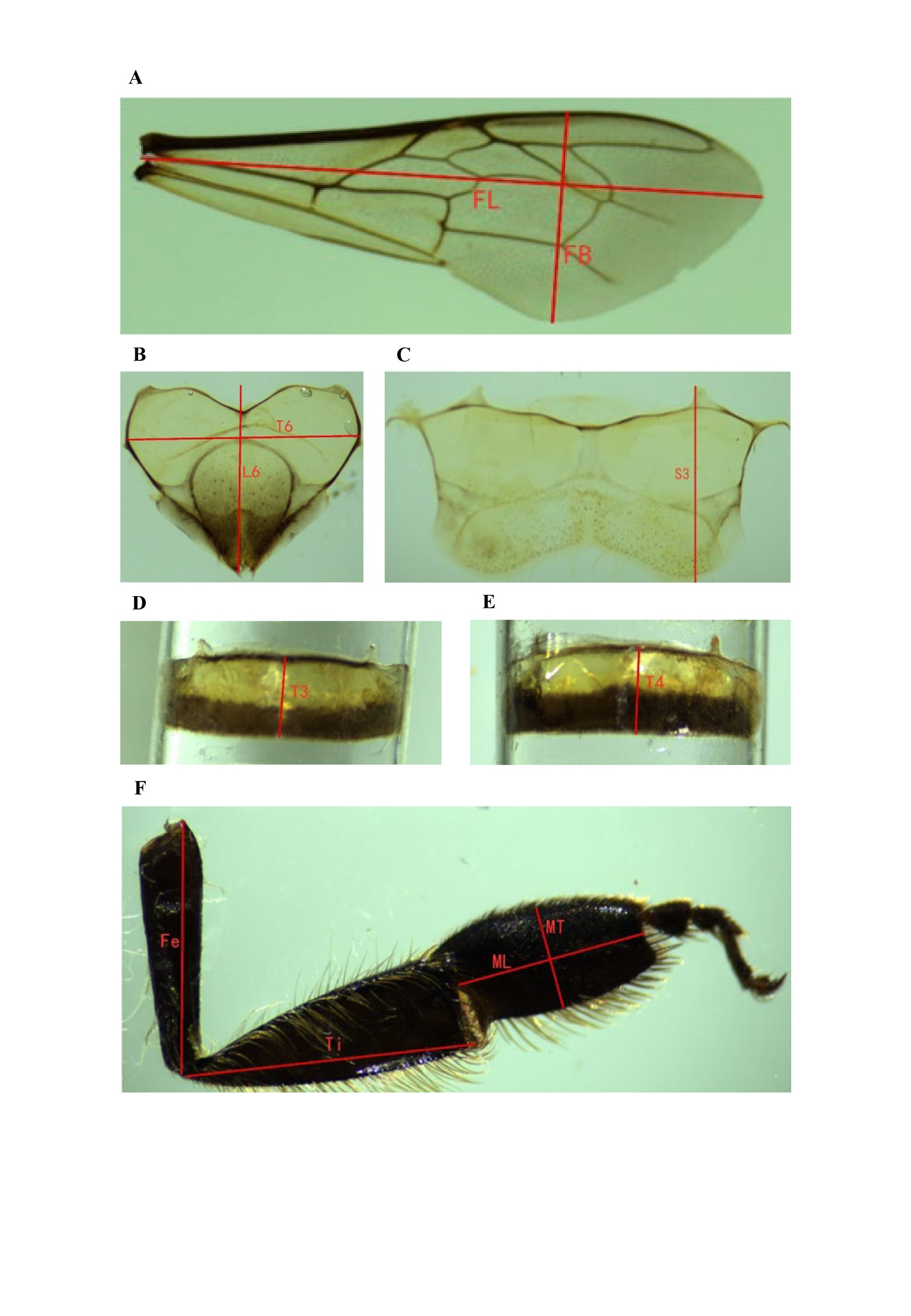


**Figure S2**. The diagram of 10 morphological indicators of *A. cerana*. (A) The right forewing length (FL) and width (FB). (B) The sixth sternum length (L6) and width (T6). (C) The third sternum length (S3). (D) The third tergum length (T3). (E) The fourth tergum length (T4). (F) The femur length (Fe), the tibia length (Ti), the basitarsus length (ML), and the basitarsus width (MT).


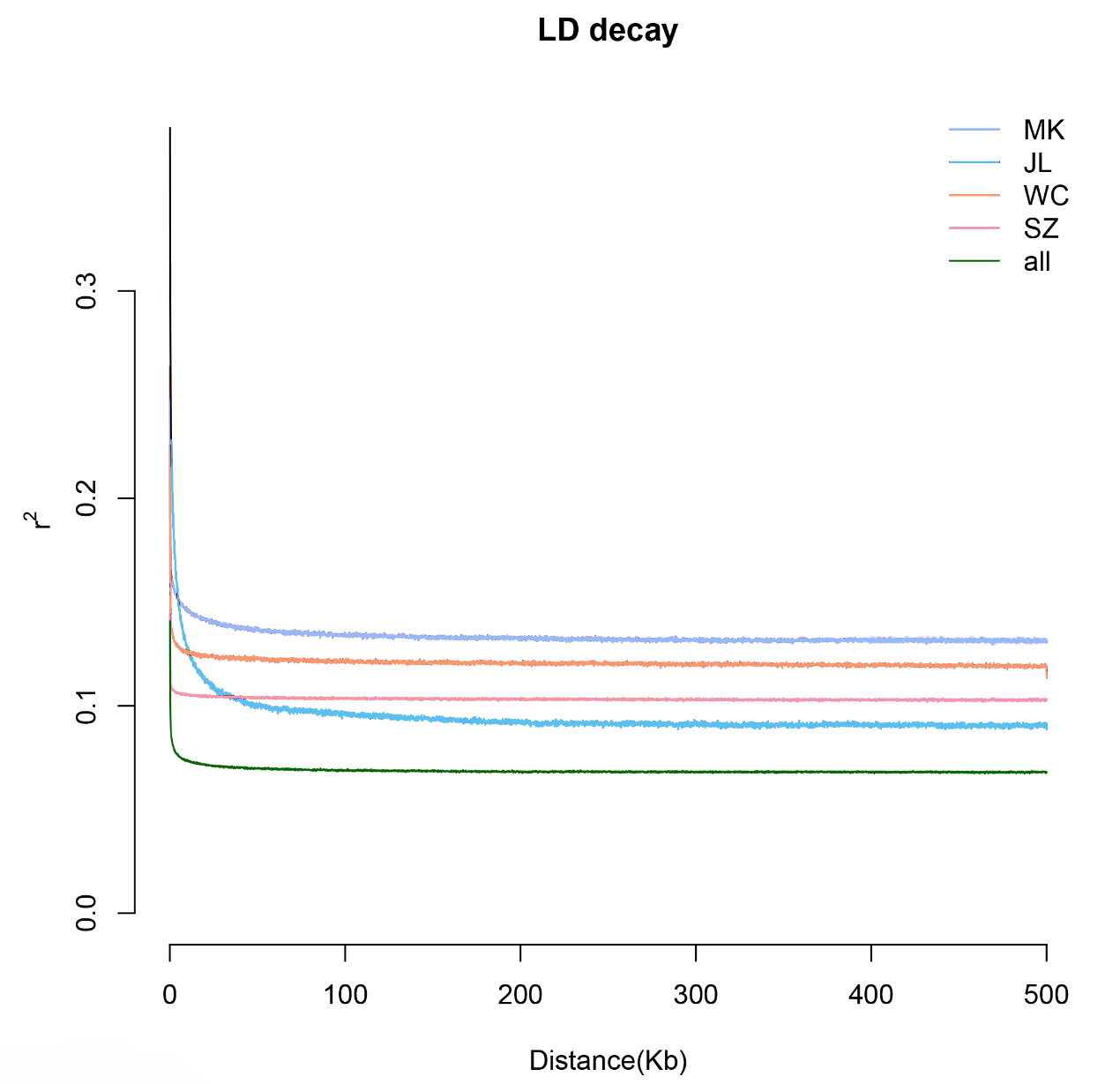


**Figure S3.** Analysis of linkage disequilibrium.


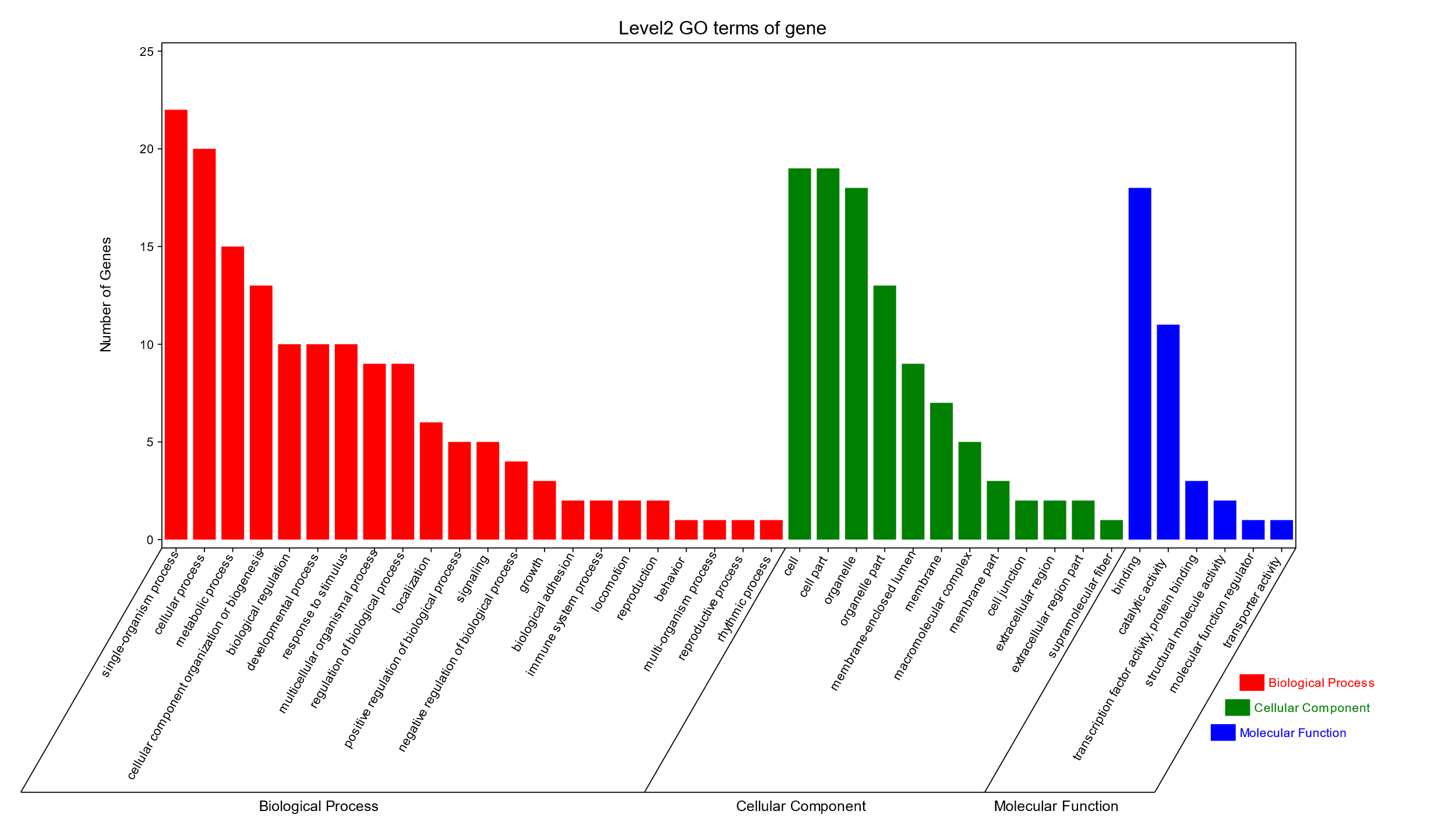


**Figure S4.** GO classification of candidate genes.


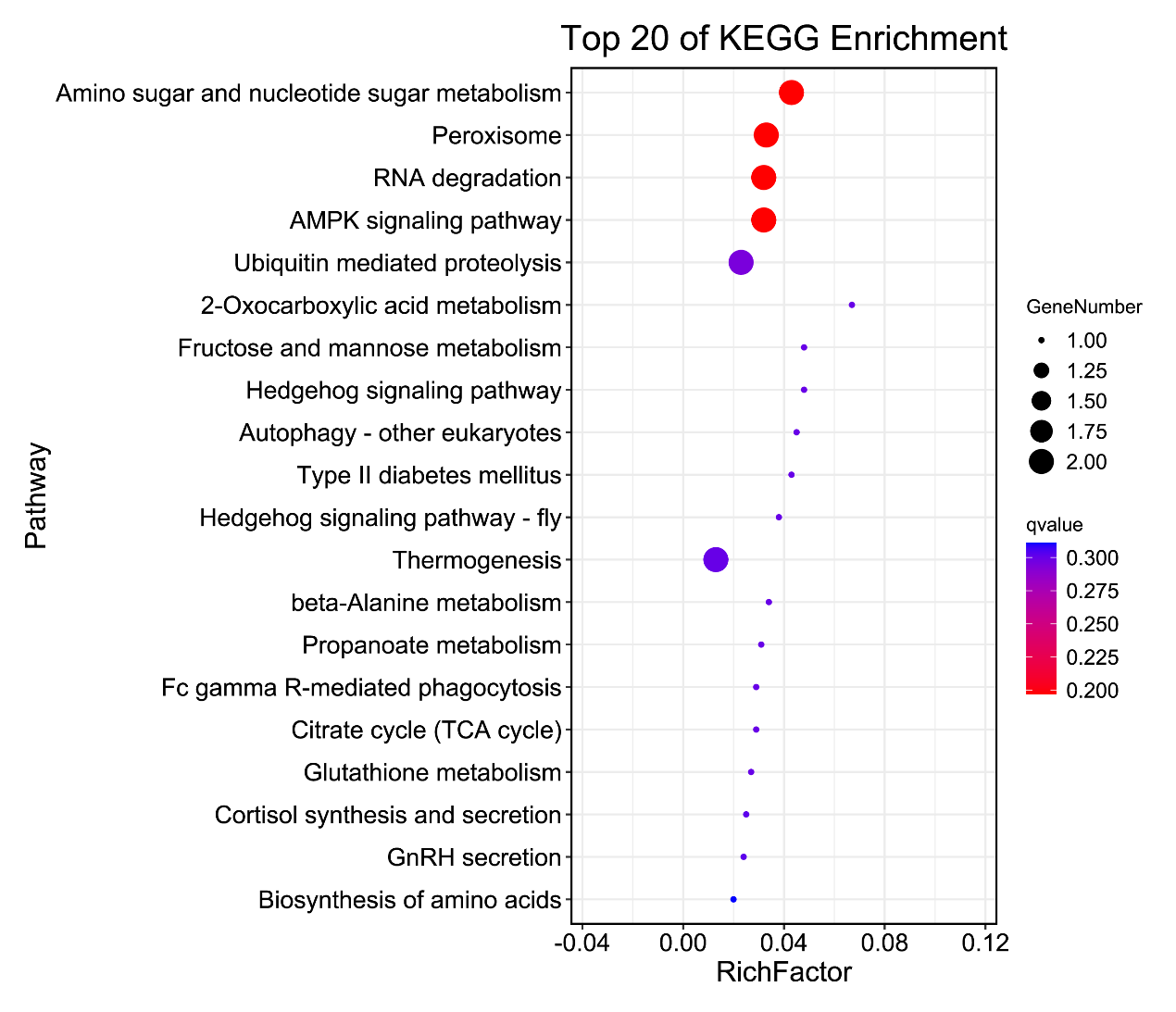


**Figure S5.** The top 20 enriched KEGG pathways.
